# Evaluation of clofazimine-bedaquiline combination as a candidate regimen for macrolide-resistant *Mycobacterium avium* complex infection

**DOI:** 10.1128/aac.01511-25

**Published:** 2025-12-19

**Authors:** Jiyun Park, Sangwon Choi, Yae Rin Jeon, Lee-Han Kim, Ju Mi Lee, Sung Jae Shin

**Affiliations:** 1 Department of Microbiology, Institute for Immunology and Immunological Disease, Graduate School of Medical Science, Brain Korea 21 Project, Yonsei University College of Medicine 37991, Seoul, South Korea; City St George's, University of London, London, United Kingdom

**Keywords:** *Mycobacterium avium *complex, clofazimine, bedaquiline, macrolide-resistance, drug combination, murine model

## Abstract

The *Mycobacterium avium* complex (MAC) is the primary cause of pulmonary disease (PD) among nontuberculous mycobacteria, presenting a significant treatment challenge on a global scale. A long-term (≥12 months) three-drug regimen, typically including a macrolide, such as clarithromycin (CLR) or azithromycin, along with rifampicin and ethambutol, is recommended. However, many patients fail to respond adequately to therapy, and some eventually develop macrolide resistance, making the disease even more difficult to treat. This highlights the urgent need for improved therapeutic strategies. Here, we investigated the efficacy of clofazimine (CFZ) and bedaquiline (BDQ), both repurposed from multidrug-resistant tuberculosis therapy, against macrolide-resistant MAC. In macrophage infection assays, both CFZ and BDQ showed significant intracellular inhibitory activity against macrolide-resistant clinical isolates, with CFZ generally exhibiting stronger effects. In a chronic murine model of MAC-caused progressive PD, substitution of CLR with CFZ and BDQ in the treatment regimen led to marked reductions in bacterial loads in both lung and spleen compared with the standard regimen, achieving up to 0.86 log₁₀ CFU reduction in lung and 2.17 log₁₀ CFU in spleen tissues. These findings demonstrate that CFZ and BDQ retain potent activity against macrolide-resistant MAC and highlight their potential as promising components of alternative treatment regimens.

## INTRODUCTION

Nontuberculous mycobacteria (NTM) are widespread opportunistic pathogens that frequently cause pulmonary disease (PD) in humans, with an increasing prevalence observed worldwide (1, 2). NTM encompass all *Mycobacterium* species, except for *Mycobacterium tuberculosis* and *Mycobacterium leprae*. The primary NTM responsible for disease are *Mycobacterium avium* complex (MAC), *Mycobacterium abscessus* complex, and *Mycobacterium kansasii* (2).

MAC, predominantly composed of *M. avium* (Mav) and *M. intracellulare* (Mi), is recognized as the most prevalent pathogen globally (3, 4). The recommended treatment protocol for individuals with MAC-PD involves the use of a macrolide, such as clarithromycin (CLR) or azithromycin, in combination with ethambutol (EMB) and rifampicin (RIF), administered for a period of at least 1 year following the achievement of negative culture conversion (1, 5). The macrolide is the key component of this regimen due to its significant correlation with *in vitro* susceptibility testing and clinical outcomes in MAC-PD (1). Along with the macrolide, EMB and RIF have been commonly included as companion drugs to reduce the risk of resistance and disease progression, although the contribution of RIF appears limited in MAC-PD (6–8). However, unsatisfactory outcomes, such as drug tolerance, macrolide resistance, and mortality, are sometimes observed (9–12). Macrolide resistance often arises from macrolide monotherapy, irregular use of EMB, and insufficient combination therapy with fluoroquinolones (13).

Macrolide resistance in MAC is acquired through point mutations in the 23S rRNA (*rrl*) gene, particularly at adenine 2058 and adenine 2059 (14, 15). This resistance is linked to unfavorable treatment outcomes and higher mortality rates in patients with MAC-PD (10, 16). However, clinical studies on the management of macrolide-resistant MAC-PD remain limited, and therapeutic options are not well established (10, 16, 17). In some cases, macrolides continue to be prescribed despite resistance, although their benefits in this context are controversial (12, 13, 17).

Given these challenges, alternative or adjunctive agents for the treatment of macrolide-resistant MAC-PD are urgently needed. Clofazimine (CFZ), which disrupts mycobacterial membranes and exerts anti-inflammatory effects, has emerged as a promising candidate for improving treatment outcomes in both patients and murine models with macrolide-susceptible MAC (18–22). Notably, CFZ may also help prevent the emergence of macrolide resistance, and recent guidelines suggest its use in severe or macrolide-resistant MAC-PD (1, 23). Bedaquiline (BDQ), an ATP synthase inhibitor approved for multidrug-resistant tuberculosis (MDR-TB), has demonstrated potent *in vitro* activity against both macrolide-susceptible and macrolide-resistant MAC strains (24–30). Consistent with these findings, recent studies have evaluated the *in vivo* anti-MAC efficacy of the CFZ and BDQ combination in murine models using reference strains (Mav ATCC 700898 and Mav 104), and a clinical study also suggested potential efficacy of this combination in patients with refractory NTM lung disease (31–33).

Despite these promising findings, the efficacy of CFZ and BDQ against macrolide-resistant MAC has not been fully investigated. In particular, their intracellular and *in vivo* activities have yet to be established. Therefore, we evaluated the activities of CFZ and BDQ using macrophage and a murine infection model with macrolide-resistant MAC clinical isolate.

## MATERIALS AND METHODS

### MAC strains and cultivation

This study utilized two reference strains, Mav ATCC 700898 and Mi ATCC 13950 (American Type Culture Collection [ATCC], Manassas, VA, USA), along with 18 clinical isolates of macrolide-resistant MAC obtained from the Samsung Medical Center (SMC; Seoul, South Korea). The clinical isolates of macrolide-resistant MAC were identified as having point mutations in the 23S rRNA at the macrolide resistance-associated region (15, 34). As previously reported, strains were grown in Middlebrook 7H9 broth (BD-Difco, Le Pont de Claix, France), containing 10% oleic acid-albumin-dextrose-catalase (OADC) at 36°C. For quantification, colony-forming units (CFUs) were counted on Middlebrook 7H10 agar (BD-Difco) (35).

### Antibiotics

CLR, RIF, and EMB were obtained from Tokyo Chemical Inc. (Tokyo, Japan), whereas CFZ was sourced from Sigma-Aldrich, Inc. (St. Louis, MO, USA). BDQ was procured from AdooQ Bioscience (Irvine, CA, USA). All antibiotics were dissolved in dimethyl sulfoxide, except for EMB, which was dissolved in distilled water and diluted in Dulbecco’s phosphate-buffered saline (DPBS; Biowest, Nuaillé, France) for *in vitro* drug susceptibility testing (DST) and intracellular activity testing. For oral administration in mice, 0.5% carboxymethylcellulose served as the vehicle.

### Animals

Six-week-old specific-pathogen-free female BALB/c mice were acquired from Orient Bio, Inc. (Sung-nam, South Korea).

### *In vitro* drug susceptibility testing

Twenty MAC strains were subjected to *in vitro* DST using the broth microdilution resazurin assay after 7 days of incubation, according to Clinical and Laboratory Standards Institute (CLSI) guideline (36). CLR resistance was defined as minimum inhibitory concentration (MIC) ≥32 µg/mL (36). All MIC values were confirmed in triplicate assays with duplicate wells.

### Intracellular anti-MAC activity testing

Murine bone marrow-derived macrophages (BMDMs) were differentiated from BALB/c bone marrow cells by culturing in Dulbecco’s modified Eagle’s medium (Biowest) supplemented with 10% fetal bovine serum (Biowest) and 10% L929 cell supernatant for 6 days. BMDMs (4 × 10^5^ cells/mL) were infected with MAC strains at a multiplicity of infection of 3 for 4 h, followed by drug treatment at the indicated concentrations in triplicate wells for 72 h, as previously described (37). To minimize vehicle-related effects, the final concentration of DMSO was adjusted to 0.1% in all drug-treated wells. Cells were then lysed with 0.05% Triton X−100, serially diluted in DPBS, and plated (four spots per well) on Middlebrook 7H10 agar containing 10% OADC. Following 1 week of incubation at 36°C, colonies were enumerated and expressed as mean CFUs ± standard deviations (SDs) per mL. Each experiment was performed independently at least twice.

### Assessment of anti-MAC effects of drug combinations in a mouse model with macrolide-resistant MAC lung infection

To evaluate the effectiveness of various treatment regimens based on EMB and RIF, 35 BALB/c mice were exposed to macrolide-resistant Mav SMC #422 through aerosol inhalation using a system from Glas-Col (Terre Haute, IN, USA). Three mice were sacrificed 1 day after infection to determine the initial infection level, which averaged 9.4 × 10^4^ CFUs in the lungs. Ten weeks after infection (pre-treatment phase [Pre-Tx]), three mice were sacrificed to assess the bacterial load before treatment. Of the remaining mice, five were assigned to the untreated infection control group (Con.), and six mice per group were allocated to each treatment regimen. Treatment commenced at the 10-week mark and lasted for 3 weeks, utilizing either standard or alternative regimens. Mice received daily doses of CLR and EMB (100 mg/kg each), RIF (10 mg/kg), CFZ (20 mg/kg), and BDQ (25 mg/kg), as described previously (21, 38–41). All drugs were mixed into a single suspension in 0.5% carboxymethylcellulose and administered simultaneously once daily by oral gavage. After 3 weeks of treatment, mice were euthanized, and lung tissues were homogenized to determine the bacterial loads.

### Statistical analysis

Data were analyzed by ordinary one-way ANOVA with Tukey’s multiple comparison test using GraphPad Prism version 9 (GraphPad Software, https://www.graphpad.com/, La Jolla, CA, USA). Statistical significance was defined as *P* < 0.05.

## RESULTS

### *In vitro* DST for CFZ and BDQ against macrolide-resistant MAC clinical isolates

To assess the *in vitro* effectiveness of CFZ and BDQ against macrolide-resistant MAC, MICs were determined for 18 clinical isolates from patients with macrolide-resistant MAC-PD and two reference strains, Mav ATCC 700898 and Mi ATCC 13950. Except for Mav SMC #411, which was isolated from a treatment-failed patient without mutations in 23S rRNA, all clinical isolates carried point mutations at positions 2058 or 2059 in domain V of the 23S rRNA gene. As expected, these mutations were associated with high-level CLR resistance, with MICs >64 µg/mL, whereas Mav ATCC 700898 and Mi ATCC 13950 showed low MICs of 0.5 and 0.125 µg/mL, respectively. In contrast, CFZ MIC values were between 0.5 and 4 µg/mL, while BDQ exhibited markedly lower MICs of 0.004 to 0.015 µg/mL, clearly indicating its far superior *in vitro* potency against macrolide-resistant MAC (Table 1).

**TABLE 1:** MICs of indicated drugs against macrolide-resistant strains^*a*^

Species	23S rRNA mutation A2058/A2059	MIC (µg/mL)		
		CLR	CFZ	BDQ
Mav ATCC 700898^*b*^	Not mutated	0.5	2	0.015
Mav SMC #397	A2059C	>64	1	0.004
Mav SMC #422	A2059G	>64	0.25	0.008
Mav SMC #1216	A2058C	>64	2	0.004
Mav SMC #411	Not mutated	>64	1	0.004
Mav SMC #417	A2058C	>64	1	0.004
Mav SMC #420	A2058C	>64	1	0.008
Mav SMC #1213	A2058T	>64	0.5	0.008
Mav SMC #1217	A2058C	>64	4	0.004
Mi ATCC 13950^*b*^	Not mutated	0.25	2	0.008
Mi SMC #400	A2058C	>64	4	0.008
Mi SMC #418	A2058G	>64	1	0.015
Mi SMC #402	A2059C	>64	2	0.004
Mi SMC #404	A2059G	>64	2	0.004
Mi SMC #407	A2059G	>64	0.5	0.004
Mi SMC #408	A2059G	>64	0.5	0.008
Mi SMC #412	A2058G	>64	1	0.004
Mi SMC #414	A2058G	>64	0.5	0.004
Mi SMC #423	A2058G	>64	0.5	0.008
Mi SMC #427	A2058G	>64	0.5	0.004

^*a*^ CLR, clarithromycin; CFZ, clofazimine; BDQ, bedaquiline; SMC, Samsung Medical Center.

^*b*^ Used as reference strains in the relevant experiments of this investigation.

### Assessment of intracellular antimycobacterial effects of CFZ and BDQ against macrolide-resistant MAC clinical isolates

**Fig 1:**
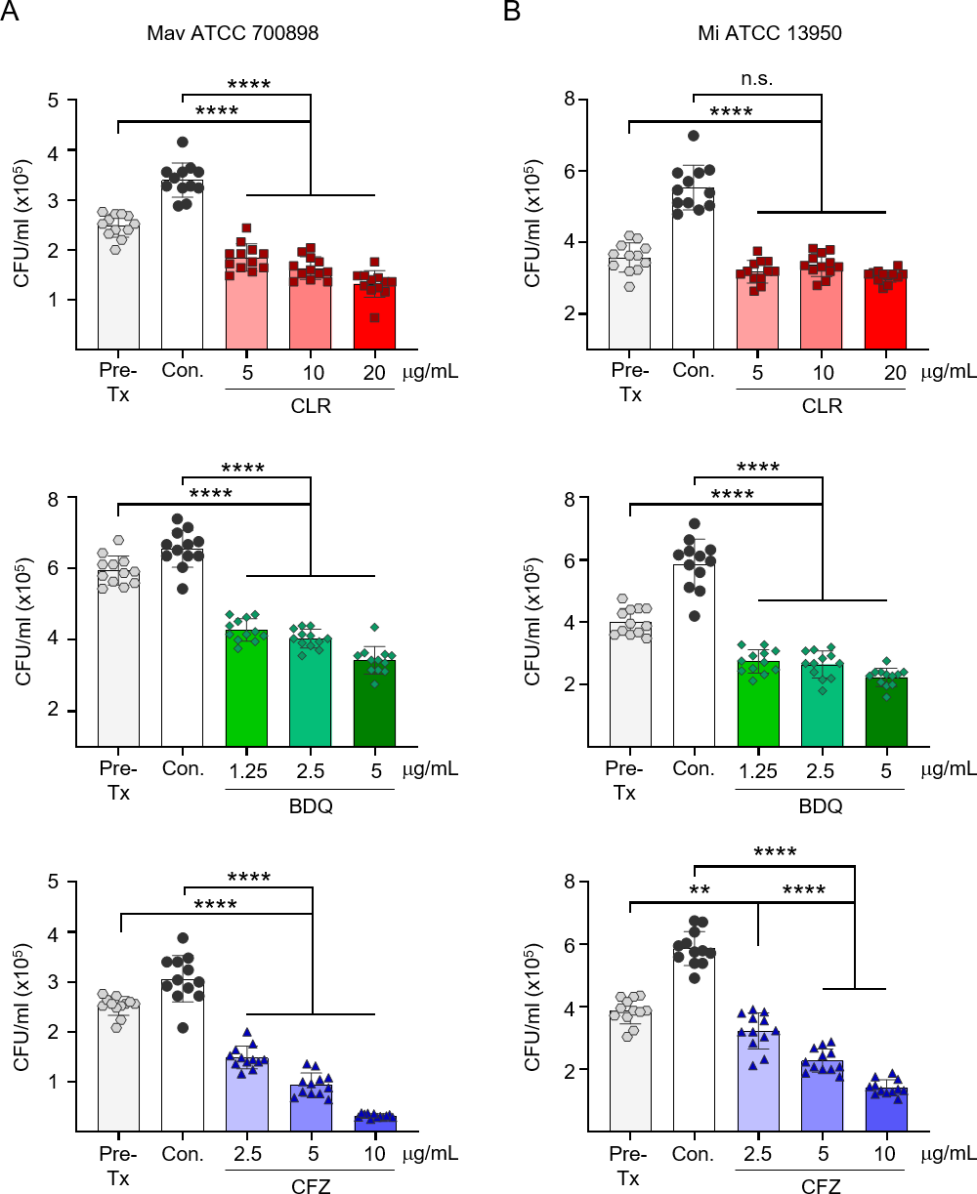
Dose-dependent intracellular activities of CLR, CFZ, and BDQ against MAC reference strains. BMDMs were infected with MAC reference strains, (**A**) Mav ATCC 700898 and (**B**) Mi ATCC 13950, and treated with increasing concentrations of CLR, CFZ, and BDQ. Bacterial survival was assessed at 72 h post-infection by colony enumeration on 7H10-OADC agar plates. Data are represented as a scatter plot with bars, where each dot presents the mean ± SD of triplicate wells. The one-way ANOVA with Tukey’s multiple comparison test was used to evaluate significance compared to Con. *P < 0.05, **P < 0.01, ***P < 0.001, ****P < 0.0001, n.s., not significant. Mav, *Mycobacterium avium*; Mi, *Mycobacterium intracellulare*; Pre-Tx, Pre-treatment (initiation of treatment); Con., untreated infection control; CFZ, clofazimine; BDQ, bedaquiline.

We then assessed the intracellular activities of CFZ and BDQ against macrolide-resistant MAC to evaluate their ability to inhibit bacterial growth within macrophages. To determine the appropriate concentrations, BMDMs were infected with reference strains and treated with increasing doses of each drug (Fig. 1). The maximum drug concentrations were selected based on previous studies, ensuring both biological relevance and minimal cytotoxicity (21, 42–44). All tested drugs effectively inhibited the growth of both reference strains, although the degree of dose dependency was less pronounced for CLR and BDQ against *M. intracellulare* ATCC 13950 (Fig. 1). This confirmed that the selected concentrations were effective and that the experimental conditions were appropriate for evaluating intracellular activity. In addition, cytotoxicity tests showed no significant toxicity of the selected drug concentration on BMDMs (Fig. S1).

**Fig 2:**
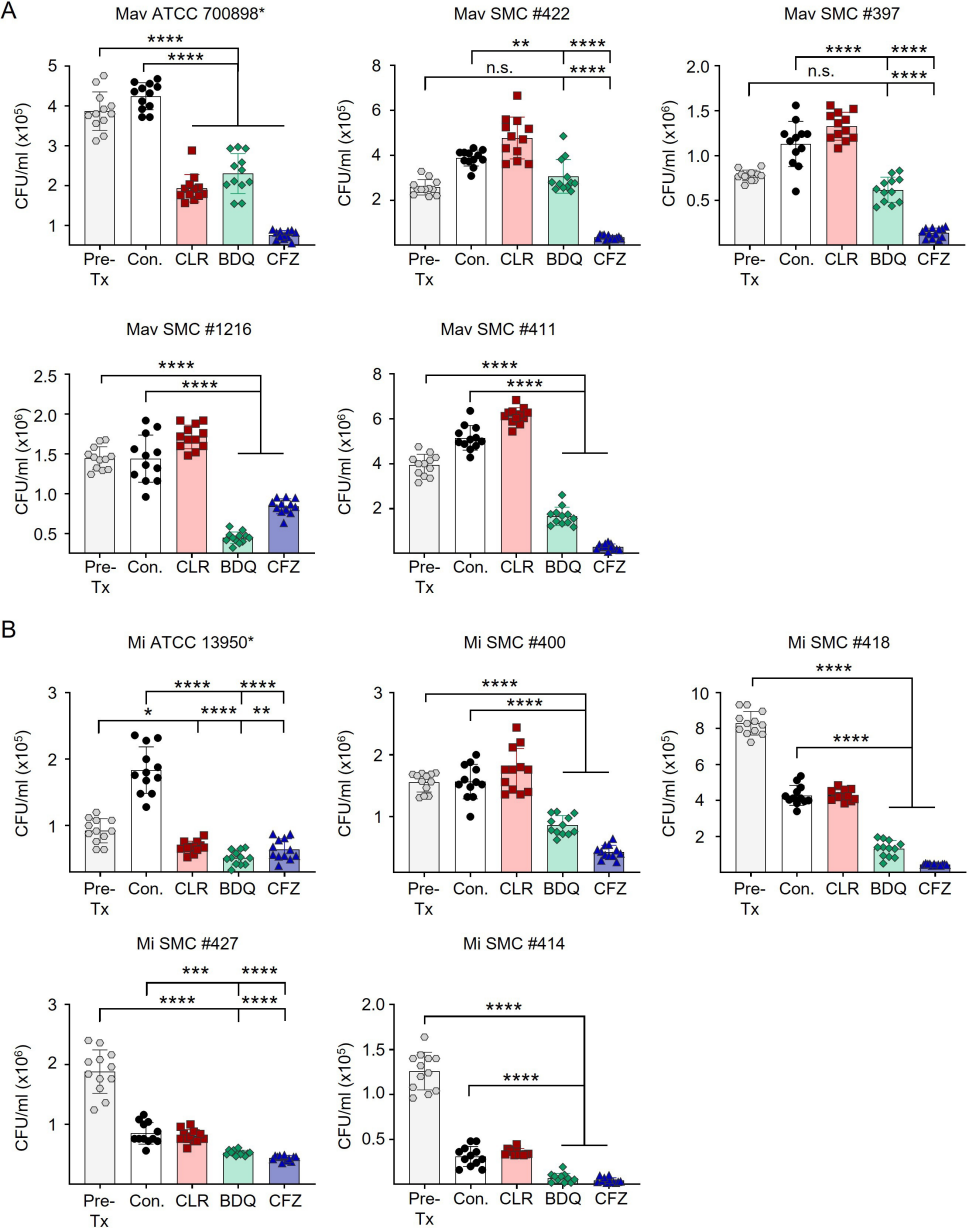
Intracellular anti-MAC activities of CFZ and BDQ against macrolide-resistant MAC clinical isolates. BMDMs were infected with macrolide-resistant MAC clinical isolates together with one reference strain for each species. (**A**) Mav strains including the reference strain Mav ATCC 700898. (**B**) Mi strains including the reference strain Mi ATCC 13950. Infected BMDMs were treated with CLR (20 µg/mL), CFZ (10 µg/mL), or BDQ (5 µg/mL). Intracellular bacterial survival was assessed at 72 h post-infection by colony enumeration on 7H10-OADC agar plates. Data are represented as a scatter plot with bars, where each dot presents the mean ± SD of triplicate wells. The one-way ANOVA with Tukey’s multiple comparison test was used to evaluate significance compared to Con. *P < 0.05, **P < 0.01, ***P < 0.001, ****P < 0.0001, n.s., not significant. Mav, *Mycobacterium avium*; Mi, *Mycobacterium intracellulare*; Pre-Tx, Pre-treatment (initiation of treatment); Con., untreated infection control; CFZ, clofazimine; BDQ, bedaquiline.

Subsequently, CLR-resistant MAC clinical isolates were used to evaluate the intracellular efficacies of the drugs. Infected BMDMs were treated with 20 µg/mL CLR, 10 µg/mL CFZ, and 5 µg/mL BDQ, and intracellular bacterial survival was evaluated at 72 h post-infection (Fig. 2). As expected, CLR treatment alone did not reduce bacterial burden against resistant strains. In contrast, both CFZ and BDQ consistently demonstrated significant intracellular inhibitory activity across all isolates. Notably, CFZ tended to exhibit stronger intracellular activity than BDQ in many cases. These results indicate that CFZ and BDQ retain their potency against macrolide-resistant MAC.

### Therapeutic potential of CFZ- and BDQ-based regimens in an experimental mouse model of macrolide-resistant MAC infection

**Fig 3:**
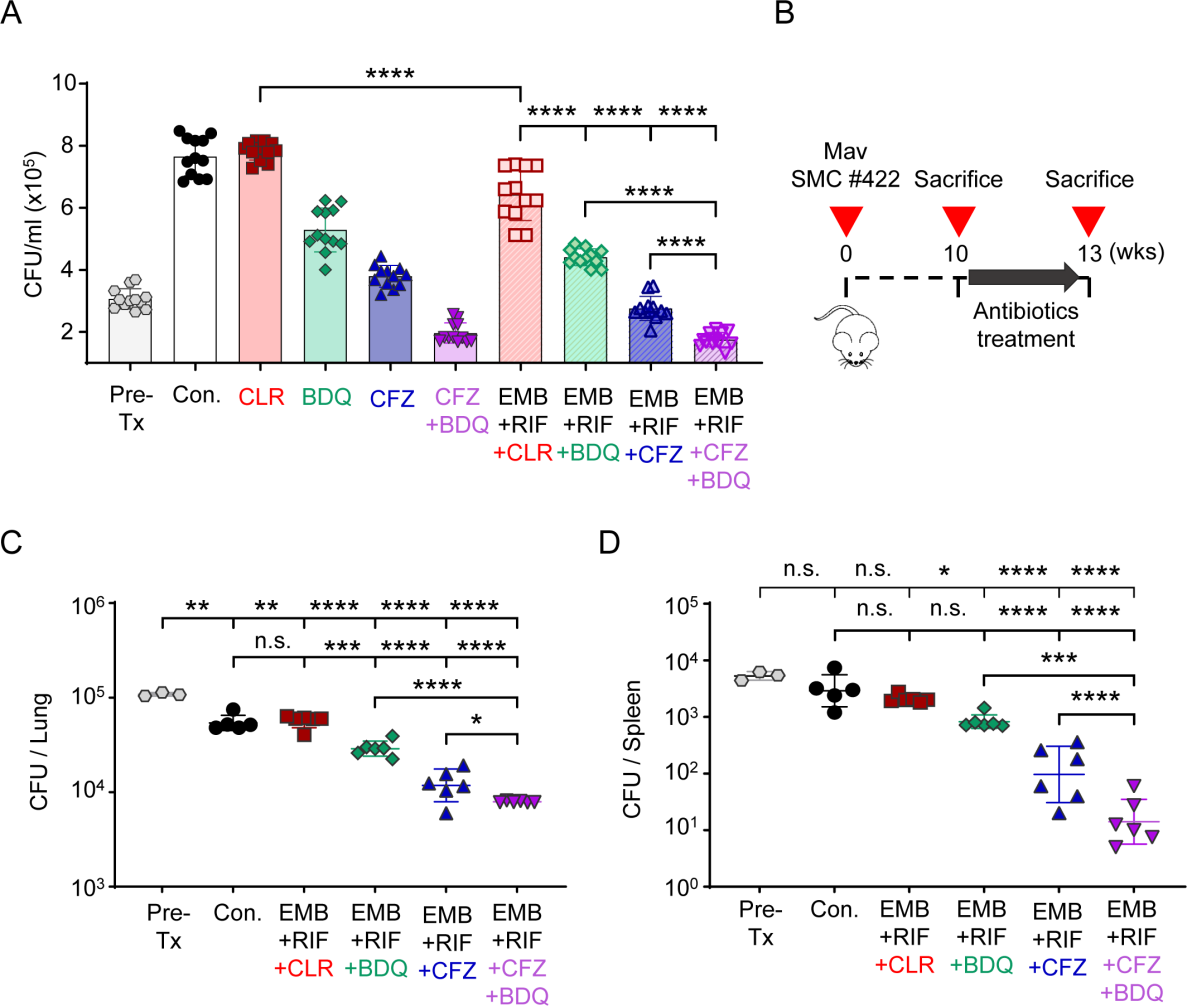
Comparative efficacy of standard and CFZ- or BDQ-containing regimens in a murine model of macrolide-resistant MAC pulmonary infection. (**A**) BMDMs were infected with Mav SMC #422 and treated with the indicated drug combinations for 72 h. The drug doses were 20 µg/mL EMB, 2 µg/mL RIF, 20 µg/mL CLR, 5 µg/mL BDQ, 4 µg/mL CFZ. Intracellular bacterial survival was assessed at 72 h post-infection by colony enumeration on 7H10-OADC agar plates. Data are represented as a scatter plot with bars, where each dot presents the mean ± SD of triplicate wells. (**B**) Experimental scheme for evaluating the anti-MAC activities of the indicated drug regimen *in vivo*. BALB/c mice were infected with Mav SMC #422 via aerosolization, and treatment was initiated at 10 weeks post-infection. (**C**) Lung and (**D**) spleen bacterial burden assessed by CFU counts after 3 weeks of treatment. CFUs were determined by plating serially diluted tissue lysates on 7H10-OADC agar plates. Data are presented as the mean ± SD. Statistical significance was calculated using one-way ANOVA followed by Tukey’s multiple comparison test. *P < 0.05, **P < 0.01, ***P < 0.001, ****P < 0.0001. n.s., not significant. Mav, *Mycobacterium avium*; Con., untreated infection control; EMB + RIF + CLR, standard regimen; EMB + RIF + CFZ, CFZ-containing regimen; EMB + RIF + BDQ, BDQ-containing regimen; EMB + RIF + CFZ + BDQ, CFZ and BDQ-containing regimen.

To compare the activity of CFZ and BDQ alone or in combination with EMB and RIF, BMDMs were infected with macrolide-resistant Mav SMC #422 and treated with the indicated regimens (Fig. 3A). CLR alone did not reduce intracellular CFU levels, whereas BDQ and CFZ each showed partial inhibition, with the CFZ + BDQ combination producing a greater reduction than either single agent. Incorporation of EMB and RIF further enhanced intracellular bacterial inhibition across all corresponding regimens, and the EMB + RIF + CFZ + BDQ combination produced the largest reduction among the tested groups. These data guided the selection of EMB- and RIF-based combination regimens for subsequent *in vivo* testing. To validate these findings *in vivo*, BALB/c mice were aerosol-infected with Mav SMC #422, and treatment was initiated 10 weeks after infection (Fig. 3B). Over a 3-week course, regimens containing CFZ and/or BDQ instead of CLR were compared with the standard regimen (EMB + RIF + CLR). Bacterial burdens in the lungs and spleens were assessed by CFU enumeration (Fig. 3C and D). The standard regimen failed to reduce bacterial loads compared with the Con. group, producing −0.02 log_10_ CFU/lung and 0.22 log_10_ CFU/spleen due to macrolide resistance. In contrast, replacing CLR with BDQ (EMB + RIF + BDQ) significantly reduced bacterial counts, showing approximately 0.28 log_10_ CFU/lung (*P* < 0.001 vs Con.) and 0.6 log_10_ CFU/spleen (not significant vs Con.) reductions. The CFZ-containing regimen (EMB + RIF + CFZ) achieved greater efficacy, resulting in reductions of 0.67 log_10_ CFU/lung and 1.55 log_10_ CFU/spleen (*P* < 0.0001 vs Con.). Moreover, the combination of BDQ and CFZ (EMB + RIF + BDQ+CFZ) further enhanced bacterial clearance, yielding overall reductions of 0.84 log_10_ CFU/lung and 2.39 log_10_ CFU/spleen (*P* < 0.0001 vs Con.). Across all regimens tested, CFZ-containing treatments produced the greatest reductions in bacterial burden, with the CFZ + BDQ combination showing the largest effect.

## DISCUSSION

This study provides the first evaluation of CFZ, BDQ, and their combination administered within EMB/RIF-based regimens against macrolide-resistant MAC in a chronic murine pulmonary infection model. In parallel, CFZ and BDQ were individually assessed in macrophages to determine their respective intracellular activities. Both agents demonstrated potent antimycobacterial activity against macrolide-resistant clinical isolates, and regimens containing these agents achieved the greatest reductions in bacterial burden in both macrophage and murine models. These findings highlight CFZ and BDQ as promising candidates for inclusion in alternative regimens targeting macrolide-resistant MAC-PD and provide a rationale for advancing these agents to clinical evaluation.

To date, several antibiotics, including RIF, EMB, fluoroquinolones, and aminoglycosides, have been employed for the treatment of patients with macrolide-resistant MAC-PD. However, their overall effectiveness, whether used alone or in combination, remains suboptimal, and clinical outcomes are generally poor (11, 12, 45–47). For example, moxifloxacin treatment failed in all patients with macrolide-resistant MAC-PD and succeeded in only 33% of those with macrolide-susceptible MAC-PD (45). Similarly, regimens combining RIF and EMB with fluoroquinolones or aminoglycosides, or those involving continued macrolide use, are largely ineffective (11, 12). Even inhaled amikacin achieved successful outcomes in only 11% of patients with macrolide-resistant MAC-PD (46). In contrast, an earlier study indicated that regimens including EMB were associated with improved sputum culture conversion compared with those without EMB in macrolide-resistant MAC-PD (47). Collectively, these findings underscore the lack of effective therapeutic options for macrolide-resistant MAC-PD and the urgent need for novel therapeutic regimens.

Given this therapeutic gap and slow pace of *de novo* drug discovery, repurposing clinically approved anti-tuberculosis agents provides a pragmatic approach to accelerate treatment development for NTM disease (30, 48). The established clinical use of CFZ and BDQ in MDR-TB offers a strong translational rationale for their evaluation in MAC-PD, as both pathogens share slow growth and similar multidrug treatment challenges (49). In this context, our study adds new evidence by demonstrating that CFZ- and BDQ-containing combinations exert significant antimycobacterial activity against MAC in a murine model of macrolide-resistant infection, thereby providing a rationale for their further clinical evaluation.

Although BDQ exhibited markedly lower MICs than CFZ *in vitro*, CFZ showed greater efficacy in both intracellular and *in vivo* models across most isolates. This apparent discrepancy may be explained by pharmacokinetic differences between the two drugs. CFZ, a highly lipophilic compound, accumulates extensively within macrophages and tissues, achieving sustained intracellular concentrations that enhance its antimycobacterial activity (50, 51). By contrast, BDQ, despite its potent inhibition of ATP synthase, displays less favorable intracellular pharmacokinetics and tissue distribution in murine models, which might limit its *in vivo* efficacy (52, 53). In our experimental design, RIF was included to reflect the current standard background regimen for macrolide-resistant MAC-PD. However, RIF markedly reduces BDQ exposure through CYP3A4 induction (54, 55), and this pharmacokinetic interaction likely contributed to the attenuated efficacy of BDQ-containing regimens. Further investigations using RIF-free combinations will be required to delineate the true therapeutic potential of BDQ against macrolide-resistant MAC.

The complementary mechanisms of CFZ (membrane destabilization) and BDQ (ATP synthase inhibition) may yield additive or synergistic activity in mycobacterial models (56, 57), which is consistent with the enhanced bacterial clearance observed with the CFZ + BDQ regimen in this study. While efficacy was assessed at a single endpoint (3 weeks), previous investigations demonstrated consistent bactericidal activity of CFZ- and BDQ-containing regimens across multiple time points in chronic Mav infection models (31) and favorable clinical outcomes in patients with refractory NTM-PD treated with CFZ + BDQ (33). These findings indicate that the effects observed at the 3-week endpoint likely reflect sustained rather than transient efficacy and further support clinical evaluation of CFZ + BDQ-containing regimens.

Nevertheless, their use requires careful consideration of well-documented tolerability issues. CFZ can cause skin discoloration and gastrointestinal disturbances (58), whereas BDQ may lead to QT interval prolongation, which could necessitate ECG monitoring in clinical settings (59). Although these adverse effects are generally manageable, they should be considered when designing future treatment regimens. In addition, as this study was conducted at the preclinical level using a single-strain-infected model, further validation across diverse strains and clinical settings is essential to establish the true therapeutic potential of these agents. Given the limited efficacy of the EMB + RIF + CLR regimen in the macrolide-resistant disease model, the efficacy of CFZ and BDQ against macrolide-resistant MAC observed in this study provides important evidence supporting their potential as key components in future treatment strategies.

## Data Availability

All data sets presented in this study are included in this article.

## SUPPLEMENTAL MATERIAL

Supplemental material aac.01511-25-s0001.docx Fig. S1; Supplemental methods.

ASM does not own the copyrights to Supplemental Material that may be linked to, or accessed through, an article. The authors have granted ASM a non-exclusive, world-wide license to publish the Supplemental Material files. Please contact the corresponding author directly for reuse.
