# Supplementary material for: Evaluation of clofazimine-bedaquiline combination as a candidate regimen for macrolide-resistant Mycobacterium avium complex infection
Source: Antimicrob Agents Chemother. 2025 Dec 19;70(2):e01511-25. doi: 10.1128/aac.01511-25 (PMC12888858; doi:10.1128/aac.01511-25)
Supplement: Supplemental material — Fig. S1; Supplemental methods. [file aac.01511-25-s0001.docx]

**Supplementary information**

**Evaluation of clofazimine-bedaquiline combination as a candidate regimen for macrolide-resistant *Mycobacterium avium* complex infection**

Jiyun Park^†^, Sangwon Choi^†^, Yae Rin Jeon, Lee-Han Kim, Ju Mi Lee* and Sung Jae Shin*

Department of Microbiology, Institute for Immunology and Immunological Disease, Graduate School of Medical Science, Brain Korea 21 Project, Yonsei University College of Medicine, Seoul, South Korea

*E-mail: [jmlee@yuhs.ac](mailto:jmlee@yuhs.ac) and [sjshin@yuhs.ac](mailto:sjshin@yuhs.ac)

**Supplemental Figure**

**
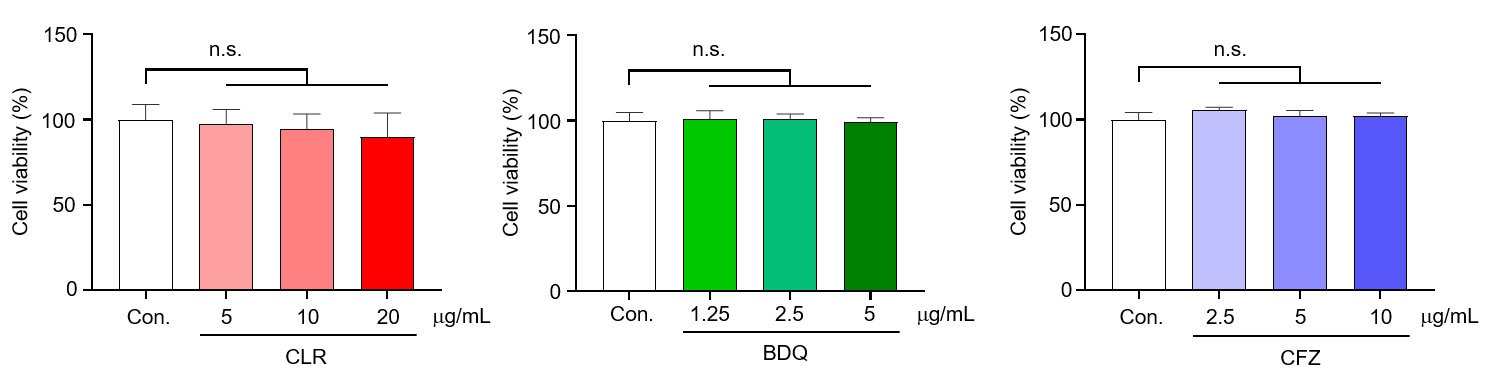
**

**FIG. S1. Evaluation of drug cytotoxicity in BMDMs.** BMDMs were infected with Mav ATCC 700898 and treated with the indicated drug concentrations for 72 h. Cytotoxicity was assessed, and results from a representative experiment of at least two independent repeats are shown. Statistical analysis was performed using one-way ANOVA followed by Tukey’s multiple comparison test. n.s., not significant; Con., untreated infection control; CLR, clarithromycin; BDQ, bedaquiline; CFZ, clofazimine.

**Supplemental Methods**

**Cell cytotoxicity test** BMDMs were seeded at a density of 4 × 10^5^ cells/mL in a 48-well plate. The indicated drugs were added to each well, starting with the highest concentration followed by serial dilutions. After 72 h incubation at 37°C in 5% CO_2,_ the culture supernatants were collected, and cell cytotoxicity was assessed using a lactate dehydrogenase activity assay kit (Sigma-Aldrich) according to the manufacturer’s instructions. Absorbance was measured at 600 nm using an Epoch microplate spectrophotometer (BioTek Instruments, VT, USA).
